# Pharmacokinetics and Metabolism of Broad-Spectrum Antivirals Remdesivir and Obeldesivir with a Consideration to Metabolite GS-441524: Same, Similar, or Different?

**DOI:** 10.3390/v17060836

**Published:** 2025-06-10

**Authors:** Darius Babusis, Cynthia Kim, Jesse Yang, Xiaofeng Zhao, Guoju Geng, Carmen Ip, Nathan Kozon, Hoa Le, Jennifer Leung, Jared Pitts, Dustin S. Siegel, Rao Kalla, Bernard Murray, John P. Bilello, Roy Bannister, Richard L. Mackman, Raju Subramanian

**Affiliations:** Gilead Sciences, Inc., Foster City, CA 94404, USA; cynthia.kim@gilead.com (C.K.); jesse.yang@gilead.com (J.Y.); xiaofeng.zhao@gilead.com (X.Z.); guoju.geng@gilead.com (G.G.); carmen.ip@gilead.com (C.I.); nathan.kozon@gilead.com (N.K.); hoa.le@gilead.com (H.L.); jennifer.leung@gilead.com (J.L.); jared.pitts@gilead.com (J.P.); dustin.siegel@gilead.com (D.S.S.); rao.kalla@gilead.com (R.K.); bernard.murray@gilead.com (B.M.); john.bilello@gilead.com (J.P.B.); roy.bannister@gilead.com (R.B.); mackmanmedchem@gmail.com (R.L.M.); raju.subramanian@gilead.com (R.S.)

**Keywords:** pharmacokinetics, remdesivir, obeldesivir, antivirals, GS-441524, GS-443902

## Abstract

RNA viruses with pandemic potential pose a significant global health risk. The adenosine nucleoside analog GS-441524 is metabolized to its active GS-443902 triphosphate metabolite to inhibit a broad spectrum of RNA viruses. Intravenous (IV) remdesivir (RDV) and oral obeldesivir (ODV) are phosphoramidate and isobutyryl-ester prodrugs of GS-441524, respectively. Following administration, both RDV and ODV show rapid and broad tissue distribution, form the same GS-443902 metabolite in target tissues, and demonstrate promising in vivo efficacy across several RNA virus infection models. In an African green monkey SARS-CoV-2 infection model, respective RDV and ODV treatments yielded similar antiviral efficacy. Here, we compare the in vitro and in vivo pharmacokinetics (PK) and metabolism of RDV and ODV to highlight both similarities and differences in their absorption, metabolism, distribution, and excretion profiles. The distinct route of administration and metabolic fate of each prodrug produced in vivo PK and metabolism profiles with differential GS-441524 to tissue GS-443902 relationships, thereby supporting alternate methods for predicting human efficacious doses. Overall, a metabolism-directed prodrug design enabled optimized delivery of the identical active GS-443902 metabolite through different routes of administration, supporting broader applications of the same nucleoside analog across an expanded spectrum of potential antiviral indications.

## 1. Introduction

In 2007 (*Outbreak*) and shortly after in 2011 (*Contagion*), Hollywood produced eerily prescient films that further blurred the line between fantasy and real life. Amazingly, within a decade of each film’s release, viruses leading to hemorrhagic fever and respiratory disease, respectively, transcended the screen. The Ebola outbreak in West Africa exploded into the spotlight early in 2014 [1]. Shortly thereafter, the global impact of COVID-19 that emerged, further substantiated the need for the scientific community to enhance the state of preparedness for Disease X [2]. The ability to rapidly respond with safe and deployable antiviral treatment options to combat endemic and pandemic threats around the world is still paramount and complementary to the availability of efficacious vaccines.

**Figure 1:**
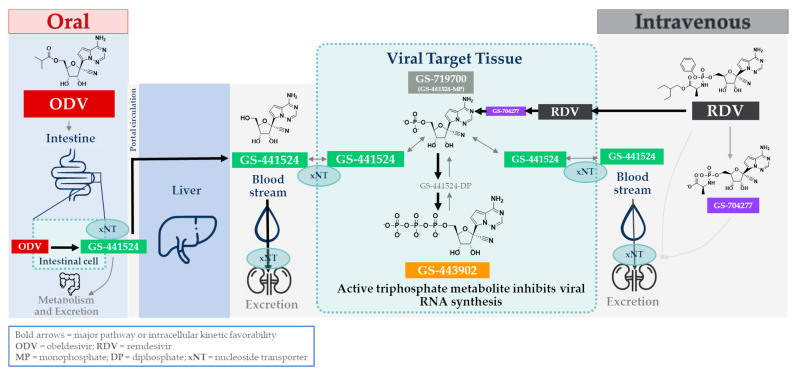
Schematic representation of the discrete administration, activation, and metabolic pathways for either oral ODV or intravenous RDV.

The C-nucleoside GS-441524 is an adenosine analog with antiviral properties that target specific processes involved in RNA virus replication [3] (Figure 1). It exerts antiviral activity as an intracellular nucleoside triphosphate metabolite (GS-443902), which then competes with natural nucleotides to selectively inhibit viral polymerases [4,5,6,7,8]. GS-441524 has broad-spectrum in vitro and in vivo activity against numerous RNA virus families [3,9,10] that warrants further profiling.

***GS-441524.*** Although GS-441524 was stable in vitro in most matrices tested, additional metabolism-focused characterization confirmed the expected physicochemical limitations of a compound with a low logD, leading to poor permeability, yet also possessing poor aqueous solubility [11]. *First*, GS-441524’s poor physical properties were anticipated to be a hurdle for oral and intracellular delivery, despite the putative advantage of facilitated uptake by nucleoside transporters [12]. *Second*, unproductive intestinal deamination pathways during absorption might be expected to further reduce the intact drug delivery absorption; GS-441524 was shown to be a weak substrate for adenosine deaminase [11]. *Third*, effective cell permeation is necessary for a nucleoside to access the viral polymerase and for intracellular metabolism to the active triphosphate; however, GS-441524 showed low membrane permeability. *Finally*, anabolism to the active GS-443902 metabolite inside tissues was found to be inefficient because of a limited first phosphorylation step, typical of nucleoside analogs [13,14]. Taken together, the poor cell permeability and inefficient first phosphorylation suggested prodrugs would be necessary to address these limitations.

GS-441524 has also demonstrated excellent efficacy in animal models [15]. The early, promising results with GS-441524, especially following the discovery of its respiratory syncytial virus (RSV) activity [10], prompted extensive efforts to refine this compound. GS-441524-related molecules were advanced through leveraging Gilead’s expertise in transforming a conceptually active, but potentially difficult to develop, chemical entity into a viable drug product [9,10]. This resulted in two prodrugs based on GS-441524: the nucleotide prodrug remdesivir (RDV) and the nucleoside prodrug obeldesivir (ODV) (Figure 1; adapted from [16]).

***Remdesivir (RDV).*** Early efforts to maximize the antiviral potential of GS-441524 leveraged nucleotide prodrug experience to enhance delivery of the GS-441524-monophosphate metabolite (GS-719700) into cells via an intermediate alaninyl metabolite (GS-704277), thereby overcoming the inefficient first anabolism step. This resulted in the discovery of the monophosphoramidate prodrug RDV as a potential RSV therapeutic [10] and later repurposed as an Ebola development candidate [9]. RDV distinguished itself as a lead candidate from an array of designed prodrugs with improved cell type-dependent in vitro antiviral potencies that were typically at least an order of magnitude better than that for GS-441524 [9,10]. In vitro metabolism studies across multiple relevant cell types also demonstrated markedly more efficient GS-443902 formation than with GS-441524 itself [17], as well as the dependence of GS-443902 concentration on improved potencies [18,19]. The utility of orally administered RDV is precluded by its instability during absorption and extensive first-pass hepatic extraction, which limits systemic exposure to RDV or any productive metabolites [10]. IV-administered RDV demonstrated excellent efficacy in numerous animal models targeting a variety of RNA viruses with pandemic potential [17,20,21,22,23,24,25]. In humans, IV-administered RDV was superior to placebos and was approved for use in COVID-19 hospitalized and high-risk non-hospitalized patients [26,27,28]. It is also being administered to patients in additional indications [29].

***Obeldesivir (ODV).*** While delivery of GS-441524-monophosphate through RDV is of proven benefit, its daily IV route of administration imposes limitations with respect to patient convenience. Although the anabolism to GS-443902 is less efficient from GS-441524 compared to GS-441524-monophosphate, GS-441524 still displays cell-culture antiviral activity in the micromolar range. Results obtained with IV administration of GS-441524 in the SARS-CoV-2-infected African green monkey (AGM) efficacy model established that high systemic exposures of GS-441524 were indeed efficacious [19]. Building from this result, the design of an oral option for the delivery of GS-441524 was thoroughly investigated and culminated in the discovery of ODV [11]. ODV is a 5’-isobutyryl ester prodrug of GS-441524 (Figure 1). Following oral administration, it extensively converts to GS-441524 in GI tract enterocytes (primary intracellular enzyme localization) and shows improved entry of GS-441524 into systemic circulation. Despite the in vitro potency and metabolism differences between RDV and either GS-441524 or ODV, similar in vivo efficacy was observed in the AGM SARS-CoV-2 model, albeit at different molar doses of each drug, reflective of the different metabolism efficiencies. More recently, ODV has demonstrated efficacy in nonclinical models across several other RNA viruses, including RSV [30], Ebola virus, and other RNA viruses with pandemic potential [25,31,32]. Furthermore, ODV is being evaluated in multiple clinical studies for several RNA virus indications.

GS-441524 is the most abundant plasma component following the administration of either IV RDV or oral ODV. As such, GS-441524 is technically classified as a major metabolite (i.e., >10% of drug-related area under the curve (AUC)), with consideration for metabolites in safety testing guidelines [33] based on steady-state plasma exposures following the clinical IV RDV or oral ODV regimen [16,34]. However, following RDV administration, GS-441524 is neither directly liberated from the prodrug in plasma, nor the component by itself for antiviral activity, nor a sole or even significant contributing entity responsible for efficient GS-443902 formation in tissues to drive efficacy, as some have postulated [35,36,37,38]. Experimental evidence demonstrating how RDV and ODV achieve similar efficacy, with distinct GS-441524 plasma PK, becomes apparent only when combining foundational aspects from both in vitro and in vivo profiling.

An array of in vitro and in vivo drug metabolism and pharmacokinetic studies was conducted, which is summarized here to present comprehensive metabolic and PK profiles of GS-441524, along with prodrugs ODV and RDV. The physicochemical and metabolic properties of GS-441524, RDV, and ODV are summarized in Table 1; select data were previously reported [9,10,11,39,40]. This explains how they were designed to achieve a similar outcome via discrete routes of administration—IV for RDV and oral for ODV (Figure 1). While descriptions of select aspects of these compounds have been previously published and/or disseminated, additional results detailed herein present a comprehensive nonclinical PK summary and holistic analysis of these antiviral therapeutics. Lastly, context is also provided on the distinct nonclinical PK-PD and efficacy data that were used to support human dose predictions for each of these prodrugs.

## 2. Materials and Methods

### 2.1. Compounds, Reagents, and In Vivo Formulations

GS-441524, ODV, RDV, GS-704277, GS-443902, and internal standards for mass spectrometry (MS)-based analyses were synthesized by Gilead Sciences (Foster City, CA, USA). [^3^H]GS-441524 and [^3^H]RDV (~25 Ci/mmol; ≥99% radiopurity), incorporating the tritium label on the C3 carbon of the 4-aza-7,9-dideazaadenosine moiety, were synthesized by Vitrax (Placentia, CA, USA). [^14^C]ODV and [^14^C]RDV (~58–59 mCi/mmol; ≥99.8% radiopurity), incorporating the ^14^C label on the 1′-cyano carbon of the ribose moiety, were synthesized by Moravek Biochemicals (Brea, CA, USA). All other chemicals, incubation plates, or reagents, unless otherwise specified, were purchased from BD Biosciences (Woburn, MA, USA), Corning (Corning, NY, USA), Sigma-Aldrich (St. Louis, MO, USA), VWR (West Chester, PA, USA), Thermo Fisher Scientific (Carlsbad, CA, USA), or Toronto Research Chemicals (North York, ON, Canada).

### 2.2. In Vitro Characterization

#### 2.2.1. Permeability and Transporter Substrate Assesment

Canine multidrug resistance 1 (MDR1)-knock-out, human MDR1-expressing, Madin–Darby canine kidney (MDCKII-cMDR1-KO) cells were maintained in Dulbecco’s modification of Eagle’s medium with sodium pyruvate and GlutaMAX, supplemented with penicillin/streptomycin and 10% fetal bovine serum in an incubator set at 37 °C, 90% humidity, and 5% CO_2_. The cells were grown to confluence on a 1 µM pore size polyester membrane in 96-well plates. The donor and receiver buffer was Hank’s Balanced Salt Solution containing 10 mM HEPES, 15 mM glucose, and 0.1% bovine serum albumin, adjusted to pH 7.4. Following initiation by the addition of test compounds to the apical side, duplicate samples were collected from the donor (0 and 120 min) and receiver (120 min) compartments into acetonitrile–methanol–water (*v*/*v*/*v*, 72:8:20) for analysis by LC-MS/MS [11]. Trans-epithelial electrical resistance values, non-specific binding, and compound instability were assessed.

GS-441524 was assessed as a potential substrate of human nucleoside (CNT1, 2, 3, and ENT1, 2, 4)-mediated transport, according to methods previously described [39]. Briefly, GS-441524 was separately incubated at 3 µM for 30 min with MDCKII-transfected cells expressing each of the listed human nucleoside transporters or control vector, in the presence or absence of a respective inhibitor. The collected samples were analyzed by LC-MS/MS for quantitation of GS-441524 uptake.

#### 2.2.2. Plasma Stability

Stabilities of RDV and its intermediate alanine metabolite GS-704277 were assessed in both human and cynomolgus monkey (cyno) plasma. First, 2 µM (final concentration) of the compound was incubated at 37 °C for up to 4 h. Serial samples collected at 6 time points throughout the incubation were added at a ratio of 1:9 (*v*/*v*) to the respective wells of a collection plate containing organic quenching solution and internal standard. The collection plates were then evaporated to dryness, reconstituted with solution for injection, and vortexed to mix; the resulting samples were injected on an LC-MS/MS system for quantitation, as described above.

#### 2.2.3. S9 Fraction Stability

S9 stability incubations were also conducted as previously described [11]. Phenylmethylsulfonyl fluoride (PMSF), a serine protease inhibitor, is typically included in gastrointestinal (GI) isolation protocols to improve their use in the assessment of highly stable compounds. However, assays were conducted in GIS9 fractions isolated without PMSF to avoid underprediction of metabolism caused by inhibition of hydrolases. Briefly, human or cyno S9 fractions (1 mg/mL; Xenotech, Kansas City, KS, USA) from either PMSF-free GI or hepatic (Hep) tissues, in a reaction buffer containing 1 mM each of ZnCl_2_ and MgCl_2_ and diluted in PBS (pH 7.4), were incubated with 2 µM ODV. Serial samples, collected at 6 time points throughout the incubation, were added at a ratio of 1:9 (*v*/*v*) to the respective wells of a collection plate containing organic quenching solution and internal standard. The plates were then vortexed and centrifuged, and aliquots of the supernatant were added to the wells of a new collection plate containing an equal volume of water. The resultant samples were analyzed by the LC-MS/MS method, as described above.

### 2.3. In Vivo Pharmacokinetic Studies

For in vivo administration in all nonclinical studies in this report, GS-441524 and [^3^H]GS-441524 were formulated as a solution at 10 mg/mL in 5% ethanol, 30% propylene glycol, 45% polyethylene glycol 300, and 20% sterile water for injection, pH 2.5. ODV and [^14^C]ODV were formulated either as a suspension at 40 mg/mL, using 0.5% methylcellulose in water, or as a solution at ~6–7 mg/mL, using 2.5% dimethyl sulfoxide, 10% Kolliphor^®^ HS-15, 10% Labrasol^®^, 2.5% propylene glycol, and 75% water, pH 6.0. RDV, [^14^C]RDV, and [^3^H]RDV were formulated as a solution at 5 mg/mL in 12% sulfobutylether-β-cyclodextrin in water, pH 3.5.

In vivo PK study design and conduct, plasma and tissue bioanalysis, and PK parameter calculations were performed as previously described [11]. Briefly, either single IV (30 min infusion) GS-441524 (20 mg/kg), RDV (10 mg/kg), or oral ODV (11.6 mg/kg) was administered to male or female cynomolgus monkeys (N = 3/group), and blood for plasma samples was serially collected over 24 h into K_2_EDTA BD Vacutainer^®^ tubes containing dichlorvos (2 mM final concentration in blood). Blood for peripheral blood mononuclear cell (PBMC) samples was also collected at 3 time points over 24 h into Na-Citrate BD Vacutainer^®^ cell preparation tubes Additionally, an extended sampling PK study was conducted following IV RDV (10 mg/kg as a 30 min infusion) administration to male cynomolgus monkeys, and blood for both plasma and PBMC samples was serially collected over 216 h, in the same manner as described above. A final study was conducted following oral ODV administration (14.4 mg/kg) in a single, portal-vein-cannulated male cynomolgus monkey. Blood from both the portal and femoral veins was serially collected for separate plasma samples over 24 h, in the same manner as described above.

#### 2.3.1. Plasma Processing

Plasma samples were maintained in chilled cryoracks or on ice until further processing to plasma via centrifugation, and the resultant samples were stored at −80 °C or on dry ice. For bioanalysis, plasma samples were extracted via protein precipitation by transferring aliquots to low-bind sample plates, followed by successive addition of organic solvent and respective internal standard working solution(s). The plates were then vortexed and centrifuged, and aliquots of the supernatant were added to the wells of a new collection plate for evaporation under nitrogen. The resultant samples were then reconstituted in acetonitrile/water (5/95 *v*/*v*), vortexed, and centrifuged; then, the supernatants were injected onto an LC-MS/MS system.

#### 2.3.2. PBMC Processing

Blood samples were maintained at room temperature until further processing to isolate PBMCs via centrifugation, as previously described [41]. Briefly, cells isolated from the plasma layer were successively washed with ice-cold saline and ammonium chloride solutions. The isolated cell pellets were stored at −80 °C or on dry ice for further processing and analysis. Intracellular metabolites were separated on a C18 column and eluted with a gradient of an ion-pair-containing mobile phase. Metabolite quantitation used standard curves prepared in the cell extract from untreated cells. In order to calculate the intracellular concentration of metabolites, the total number of cells per sample was counted using an established DNA quantitation method [42] and a PBMC intracellular volume of 0.2 pL/cell.

#### 2.3.3. Tissue Collection

Lung samples were collected in a terminal study and later analyzed in the same manner as previously described [19]. Briefly, tissue samples were harvested under deep plane anesthesia and immediately flash-frozen in liquid nitrogen. The samples were maintained either at −80 °C or on dry ice until and during processing. The bioanalytical method was as above for PBMCs.

#### 2.3.4. Pharmacokinetic Data Analysis

Noncompartmental analysis was performed to compute the descriptive PK parameters using Phoenix WinNonlin^TM^ software (v8.4.0.6172; Certara, L.P., Radnor, PA, USA). Areas under the curve were calculated using the linear trapezoidal-linear interpolation method.

#### 2.3.5. Radiolabeled Metabolism, Excretion, and Tissue Distribution

RDV: The absorption, distribution, metabolism, and excretion (ADME) of radioactivity were determined after the administration of a single intravenous dose of [^14^C]RDV (10 mg/kg; 100 µCi/kg) to male Sprague Dawley (non-pigmented) and intact Long Evans (pigmented) rats. Blood was collected for PK analyses (N = 3/time point) at selected times through 168 h post-dose. The elimination of radioactivity in urine and feces after intravenous dosing to intact non-pigmented rats (N = 3/group) was determined through 168 h post-dose. The radioactivity in tissues collected from all rats was determined by quantitative whole-body autoradiography (QWBA) at selected times through 168 h post-dose. Plasma and excreta for metabolism and tissues for QWBA were prepared as reported previously [43]. The ADME following IV [^14^C]RDV administration in humans was reported previously [44].

ODV: The absorption, distribution, and excretion of radioactivity were determined in male and female rats following a single administration of [^14^C]ODV. A single dose of [^14^C]ODV (200 mg/kg; 200 µCi/kg) was administered via oral gavage to Wistar Hanover (non-pigmented) and Long Evans (pigmented) rats. Blood was collected for PK analyses (N = 3/time point) at selected times through 168 h post-dose. The elimination and excretion of radioactivity in urine, feces, and expired air after oral dosing to non-pigmented rats (N = 3/group) were determined through 168 h post-dose. The radioactivity in tissues (N = 1/time point) collected from all rats was determined by QWBA through 336 h post-dose. The ADME following oral [^14^C]ODV administration in humans was reported previously [16].

Microautoradiography (MARG): The distribution of radioactivity in the lungs and trachea of Sprague Dawley rats (N = 3/group) was determined following a single IV administration of either [^3^H]GS-441524 or [^3^H]RDV. Formulated solutions were administered as an infusion over 30 min via a lateral tail vein, at a nominal dose of 10 mg/kg (3700 µCi/kg). Samples of lung and trachea were harvested from animals that were sacrificed 1 h after completion of the infusion period. The lungs were divided, and a full lobe was freeze-embedded into a single block of Tissue-Tek^®^ optimal cutting temperature compound in a manner that allowed longitudinal sections (anterior to posterior lung). The trachea samples were sub-divided into cross-sections and a longitudinal section (where possible). Tissue sample blocks were maintained at approximately −80 °C, wherein the tissue sample blocks were placed in a cryomicrotome (Leica CM3050, maintained at approximately −20 °C with an operating region temperature of approximately −11 °C). Replicate 5 μm sections were cut from each sample block—2 levels from each lung sample in a longitudinal orientation (anterior to posterior lung)—and each trachea sample was sub-divided into cross-sections, including a longitudinal sample, where possible. The sections were thaw-mounted under safe-light conditions onto microscope slides pre-coated with photographic nuclear emulsion. After autoradiographic exposure (for 7, 21, and 49 days), the slides were photographically processed and histologically stained. The distribution of radioactivity was evaluated using light microscopy in a qualitative manner.

## 3. Results

### 3.1. In Vitro Characterization

Additional profiling of the RDV-intermediate, alaninyl metabolite GS-704277 (depicted in Figure 1), which is observed both in plasma and cells [17], is presented in Table 1. Compared to the hydrophilic nucleoside analog GS-441524, prodrug optimizations by masking either the free glycoside 5’-hydroxyl group in ODV or both free phosphate hydroxyl moieties of its monophosphate nucleotide in RDV yielded directional improvements in solubility and permeability. Apparent cell permeability (MDCK, A-to-B) increased approximately 10-fold with RDV relative to GS-441524 and allowed for enhanced delivery and accumulation of intracellular GS-443902, as evidenced by its improved cellular potency [10]. On the other hand, ODV was designed with higher solubility and permeability, with a focus on improving intestinal absorption. Despite the ester prodrug being inherently unstable to intestine and hepatic esterases, the modification nevertheless resulted in enhanced GS-441524 oral bioavailability across several nonclinical species [11].

#### 3.1.1. Plasma Stability

**Figure 2:**
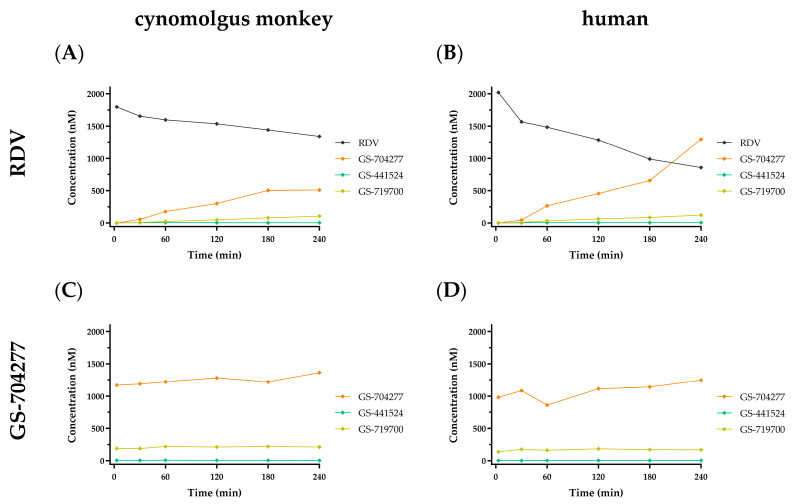
In vitro stability profiles in cynomolgus monkey and human plasma following a 2 µM incubation with either RDV (**A**,**B**) or GS-704277 (**C**,**D**) over 240 min. RDV in black; GS-704277 in orange; GS-719700 in tan; GS-441524 in green. Data are shown as the mean concentration of 2 replicates taken at each time point from a single incubation.

GS-441524 was previously shown to be stable when incubated in vitro with either cynomolgus monkey or human plasma [11]. RDV was moderately less stable (half-life; T_1/2_ = 385 and 78 min, respectively) and formed the alaninyl intermediate GS-704277 over time, presumably because of the action of plasma hydrolases (Figure 2A,B, respectively). Separately, GS-704277 itself was confirmed to be stable in both matrices, showing no degradation over the 240 min incubation (Figure 2C,D, respectively). While trace to very low-level signals were observed in the MS channel, attributed to GS-441524-monophosphate (GS-719700), during GS-704277 incubations, there was no time-dependent formation, so this may likely reflect a contaminant in the dose solution. As expected, no appreciable GS-441524 formation was observed following incubations with either RDV or GS-704277 in both cynomolgus monkey and human plasma. This is due to the acellular composition of the plasma matrix and, hence, a lack of the enzymatic machinery necessary to cleave the phosphoramide bond in the alaninyl intermediate GS-704277.

#### 3.1.2. Gastrointestinal and Hepatic S9 Fraction Stability

**Figure 3:**
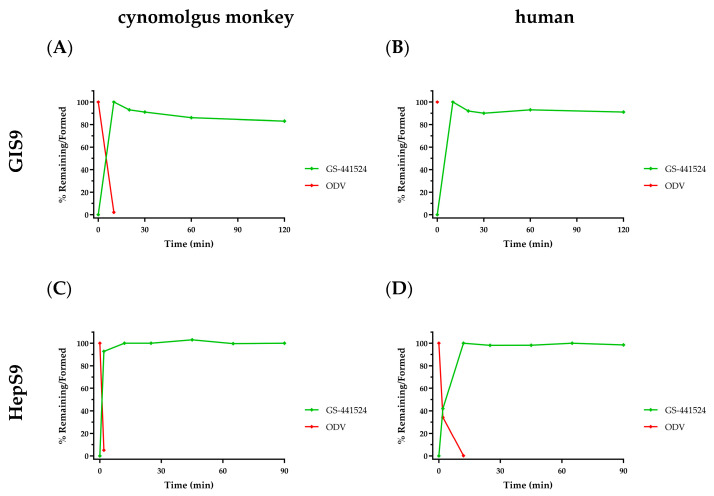
In vitro metabolism profiles following ODV incubation (2 µM) with PMSF-free S9 fractions from either cynomolgus monkey (panels (**A**,**C**)) or human (panels (**B**,**D**)) GI and Hep cells, respectively, over 120 or 90 min. ODV depletion is denoted in red and concomitant GS-441524 formation in green. Data are shown as the mean percent remaining or formed, in 2 replicates at each time point from a single incubation.

As previously reported, GS-441524 itself was stable in vitro in all matrices and enzyme preparations evaluated [11,44]. GI and hepatic S9 subcellular fractions represent the first intracellular milieu ODV encounters during oral absorption. ODV stability was expectedly poor in GI and Hep S9 fractions from cynomolgus monkeys (Figure 3A,C, respectively) and humans (Figure 3B,D, respectively). ODV largely and consistently exhibited complete metabolism by the first sampling time point following incubation initiation, with concomitant and quantitative formation of GS-441524 observed in all incubations. The in vitro ODV data suggest that because of its poor stability in intestinal and hepatic S9, it is unlikely to remain intact and is therefore expected to be completely metabolized in vivo prior to reaching systemic circulation (Section 3.2.5). Additionally, RDV was also unstable in hepatic S9 (Table 1; ref. [10]), which, at the time, suggested oral delivery was not feasible since extensive first-pass extraction would prevent sufficient intact RDV from reaching systemic circulation. As previously noted, intact RDV is necessary to deliver the monophosphate directly into cells, as designed. Utilizing IV administration as the conduit means many tissues (e.g., circulating PBMCs, lung, etc.) see intact nucleotide prodrug prior to the liver.

#### 3.1.3. Transporter Phenotyping

RDV was found to be a substrate for both OATP1B1 and P-gp (Table 1) in a screen for drug–drug interaction potential [44]. However, given its IV route of administration and enhanced permeability, the identified transporters were not anticipated to impact RDV’s effectiveness in reaching the primary indication target tissues. For ODV, the initial transporter evaluation focused on those anticipated to potentially impact oral absorption, as its noted intestinal instability would limit the influence of other transporters. ODV was a substrate for both BCRP and P-gp transporters, although any efflux during absorption would be limited by high local concentrations and its passive permeability. GS-441524 was also a substrate for P-gp. Subsequently, directed evaluation also confirmed the published findings [45] that GS-441524 is a substrate for both equilibrative (ENT1/2) and concentrative (CNT3) nucleoside transporters, which likely enhance its, albeit still limited, permeability across cell membranes.

### 3.2. In Vivo Pharmacokinetics

#### 3.2.1. Pharmacokinetics Following IV RDV Administration

**Figure 4:**
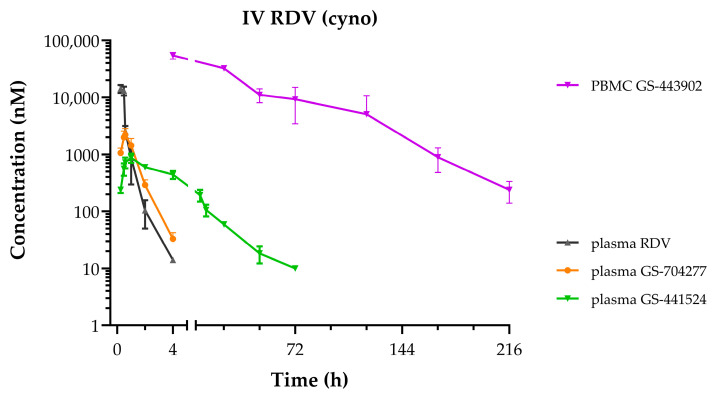
Plasma and PBMC concentration–time profiles following a single administration of IV RDV at 10 mg/kg as a 30 min infusion to cynomolgus monkeys. RDV in black, GS-704277 in orange, GS-441524 in green, and GS-443902 in purple. Data represent the mean ± SD from N = 3 monkeys.

Following the administration of RDV as a 30 min IV infusion at 10 mg/kg in cynomolgus monkeys, the PK profiles for plasma and PBMC metabolites (Figure 4) were notably similar to those observed following the administration as a slow-bolus IV injection to rhesus monkeys [17]. The mean plasma and PBMC PK parameters reported in Table 2 also depict a consistent metabolite profile in monkeys, with rapid appearance and transient exposures to both RDV and its intermediate alanine metabolite, GS-704277. Mean maximal GS-443902 concentrations in PBMCs were observed at 4 h post-dose, which then slowly declined with an effective intracellular half-life of 29 h. Of note, in contrast to the GS-441524 plasma profile described above when administered IV or orally as ODV, a more persistent exposure to low levels of GS-441524 was observed following IV RDV, with an estimated terminal elimination half-life that more closely approximates that of GS-443902 in PBMCs.

#### 3.2.2. Plasma Pharmacokinetics Following GS-441524 Administration

**Figure 5:**
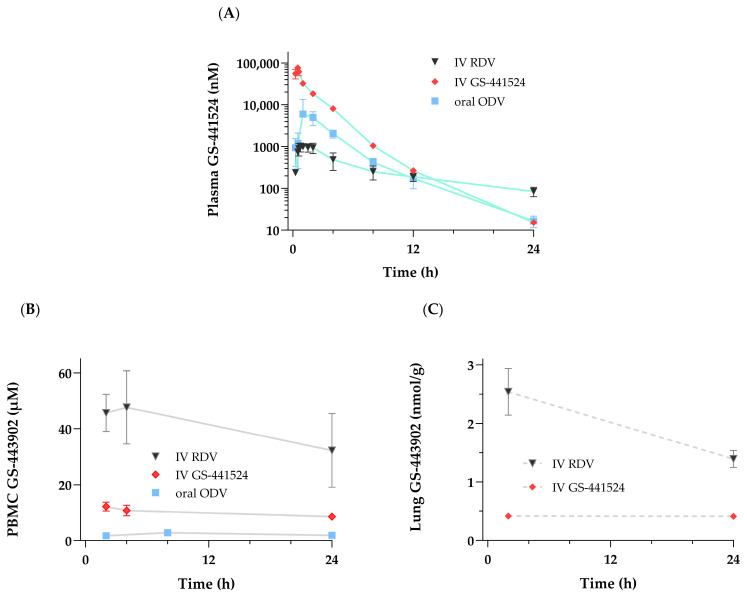
Studies were conducted following a single administration of either RDV [10 mg/kg] or GS-441524 [20 mg/kg] as a 30 min IV infusion or oral ODV [11.6 mg/kg] to cynomolgus monkeys. Concentration–time profiles over 24 h of (**A**) GS-441524 in plasma and (**B**) GS-443902 in PBMCs from all three groups. Concentrations at 2 and 24 h of (**C**) GS-443902 in the lung were only obtained from the IV-administered RDV or GS-441524 groups. Profile data are shown as mean ± SD, N = 3 monkeys except as noted. N = 2 for 2 h lung collections following IV GS-441524.

GS-441524 plasma PK was determined following intravenous administration in BALB/c mice, Sprague Dawley rats, ferrets, beagle dogs, and cynomolgus (Figure 5A) and African green monkeys. Consistent with previous reports [11,39], GS-441524 exhibited low to moderate clearance, a moderate volume of distribution, and a relatively short estimated terminal elimination half-life in plasma across species. Following oral administration, GS-441524 was rapidly absorbed and then cleared from systemic circulation, with similar estimated terminal elimination half-lives (1.4 to 4.2 h) as those observed following IV administration (0.7 to 3.4 h). However, oral bioavailability across species was highly variable and lowest in cynomolgus monkeys (F ~ 5%).

#### 3.2.3. Plasma Pharmacokinetics Following ODV Administration

Following oral administration, ODV showed rapid and extensive conversion to GS-441524 pre-systemically, as previously described [11]. The GS-441524 plasma PK profile was expectedly indistinguishable whether administered itself or as ODV, with a T_max_ typically achieved by 2 h post-dose and then rapidly eliminated (Figure 5A). Administered as a solution formulation, ODV improved GS-441524 bioavailability in cynomolgus monkeys by nearly an order of magnitude compared to the administration of the nucleoside itself [11].

#### 3.2.4. Tissue Pharmacokinetics

To provide context and relation to the intracellular PK profile of GS-443902 shown for PBMCs (Figure 5B) and lungs (Figure 5C), a comparative graph highlighting differences in exposure and the previously noted GS-441524 T_1/2_ in plasma, following the administration of either GS-441524 itself or ODV, compared to RDV, is shown in Figure 5A, and PK data are summarized in Table 3. Compared to a lower molar equivalent dose of IV RDV, higher GS-441524 plasma C_max_ values (3- to 13-fold) were associated with markedly lower mean GS-443902 concentrations in both the PBMC and gross lung samples following IV GS-441524 or in PBMCs following oral ODV. Notably, GS-443902 concentrations following IV GS-441524 and oral ODV administrations remained constant over 24 h when compared to those following IV RDV. This observation translates to a marked difference in C_24h_ tissue GS-443902-to-plasma GS-441524 AUC ratios (>20-fold in both the PBMC and lung samples) between molar-dose-adjusted IV RDV and either IV GS-441524 or oral ODV (Table 3). Additionally, following IV RDV (as detailed in Section 3.2.1), terminal elimination T_1/2_ for plasma GS-441524 or for either the PBMC or lung intracellular samples could not be accurately calculated because of the shorter collection interval (24 h) in this set of studies. Overall, the results demonstrated that once formed, GS-443902 persistence in tissues was independent of the administered compound/route.

#### 3.2.5. In Vivo ODV Characterization of Absorption and Metabolism

**Figure 6:**
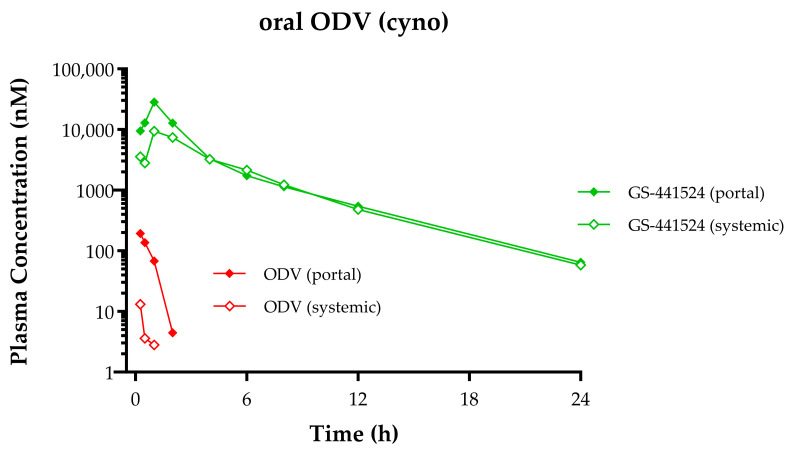
ODV and GS-441524 plasma concentration–time profiles following a single oral administration of ODV [14.4 mg/kg] to a portal-vein-cannulated cynomolgus monkey. Green lines depict GS-441524 concentrations, and red lines depict ODV. Portal vein concentrations are denoted by closed symbols, and systemic concentrations are denoted by open symbols.

The in vivo fate following oral administration of ODV and conversion to GS-441524 was studied in a portal-vein-cannulated cynomolgus monkey model. Consistent with in vitro observations in GIS9 and HepS9 fractions, the PK profile (Figure 6) following orally administered ODV confirmed that nearly complete metabolic conversion to GS-441524 occurred during absorption, likely in the intestinal tract enterocytes. Plasma ODV and GS-441524 C_max_ values in both portal and systemic circulation were observed at 0.25 and 1 h. A comparison of plasma ODV exposures from portal and systemic circulation indicated near complete hepatic extraction of any intact ODV that was absorbed. A similar comparison of plasma GS-441524 exposures yielded an apparent hepatic extraction ratio of 0.41 in this single monkey, higher than what was predicted (0.08) from scaling in vitro hepS9 data.

#### 3.2.6. In Vivo Tissue Distribution

**Figure 7:**
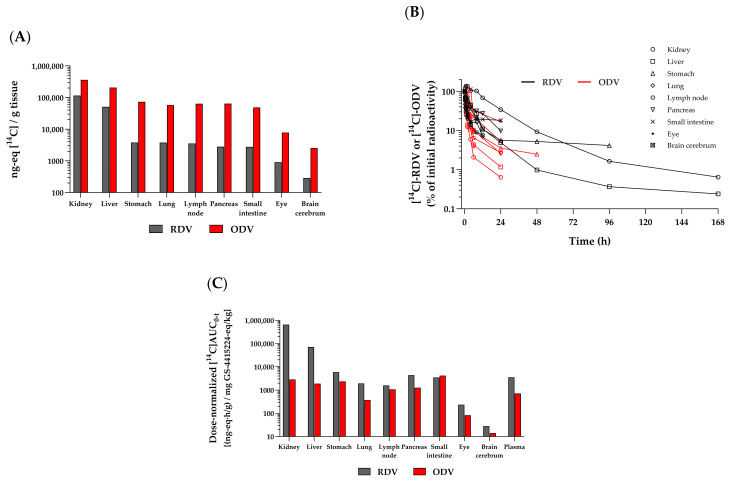
Representative radioactivity profiles in select tissues from rats: (**A**) at 0.167 h following IV [^14^C]RDV (10 mg/kg, 100 µCi/kg; in black) or at 0.25 h following oral [^14^C]ODV (200 mg/kg, 200 µCi/kg; in red) administration. (**B**) Normalized (to 0.25 h) radioactivity–time profiles from the same corresponding tissues. Hash marks at the end of each time profile denote the last measurable time point in that tissue. The last collection time points were 168 h (RDV) and 336 h (ODV). (**C**) Dose-normalized (to GS-441524 molar equivalents) AUC_0–t_ in the same corresponding tissues.

A more complete understanding of the in vivo distribution properties of RDV and ODV was assessed by QWBA of drug-associated radioactivity. Following the administration of either IV [^14^C]RDV or oral [^14^C]ODV to rats, a similarly rapid (both by 0.25 h) and broad distribution of drug-associated radioactivity throughout the body was observed (Figure 7A). The tissues highest in radioactivity were the kidney, liver, stomach, lung, lymph node, pancreas, and small intestine. IV [^14^C]RDV showed broad distribution across key tissues, with the majority of the radioactivity in the liver and kidney, which is not unexpected given that phosphoramidate prodrugs are known to be readily broken down in the liver and kidney (e.g., Sofosbuvir, etc.), and any resultant polar metabolite(s) released into circulation are predominantly eliminated renally. A low level of radioactivity, relative to more readily exposed tissues, was observed in blood compared to tissue barrier-restricted sites, such as the eyes, brain, and testes. A more pronounced persistence of higher levels of detectable radioactivity was observed in vivo across tissues following [^14^C]RDV administration (black bars/lines) than with [^14^C]ODV (red bars/lines; Figure 7B). While [^14^C]RDV-related radioactivity was still observed in several tissues at 168 h post-dose, little [^14^C]ODV-related radioactivity was detected after 24 h, with only low levels in the stomach at 48 h. When molar dose equivalents were normalized, the total tissue exposures calculated through the last measurable time point ([^14^C]AUC_0–t_; Figure 7C) further highlighted the increased persistence of [^14^C]RDV-related radioactivity across all but one of the representative tissues, relative to that from [^14^C]ODV administration. This is despite both compounds being labeled in the same manner. Expectedly, the only tissue showing a higher exposure to [^14^C]ODV-related radioactivity was the small intestine.

**Figure 8:**
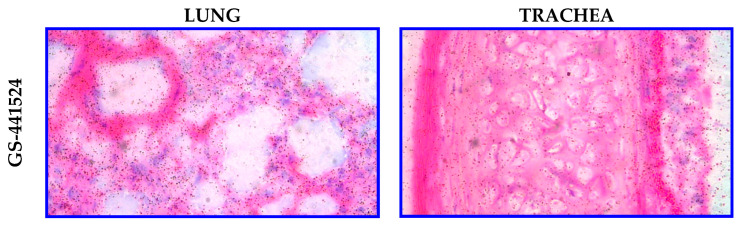

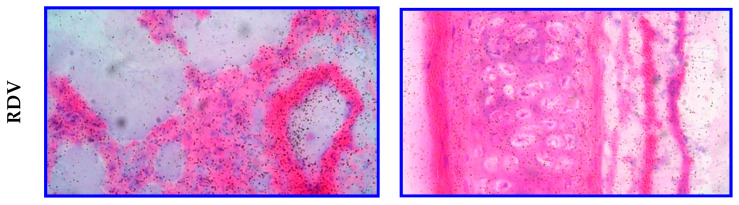
Representative microautoradiographs taken from lung and trachea tissues in Sprague Dawley rats at 1 h following a 30 min IV infusion administration of either [^3^H]GS-441524 (3700 µCi/kg) or [^3^H]RDV (3700 µCi/kg).

In a separate study, either [^3^H]RDV or [^3^H]GS-441524 was administered IV (10 mg/kg) via 30 min infusion in Sprague Dawley rats to discern potential lung and trachea tissue distribution differences using qualitative MARG (Table 4). After intravenous infusion of either compound, there was no discernible difference in the distribution of radioactivity associated with the compounds. Low to moderate levels of radioactivity associated with the investigated regions (bronchioles, alveolar wall, and blood vessels) and low levels with the alveolar sac were observed (Figure 8). The investigated regions of the trachea (adventitia, cartilage, perichondrium, submucosa, lamina propria, basement membrane, epithelium, and smooth muscle) of all animals dosed with either compound had low levels of associated radioactivity. Most notably, the MARG data showed RDV or GS-441524-derived radioactivity reaches cells in the target tissue—for example, the respiratory tract for SARS-CoV-2 or RSV indications (Table 4).

#### 3.2.7. In Vivo Metabolism and Excretion

Total mean [^14^C] dose recovery was greater than 90% following either IV RDV or oral ODV in rats and also in humans [16,44]. Following IV RDV in rats, as expected and despite including dichlorvos, no intact RDV was observed in AUC-pooled plasma samples because of the high expression levels of rodent plasma esterases [20], with approximately 70% of radioactivity as GS-441524 and 20% accounted for by GS-704277, as major circulating metabolites. In humans, 17% of the total radioactivity in plasma was accounted for by intact RDV and ~53% by GS-441524 [44]. Following oral ODV in both rats and humans, GS-441524 accounted for >97% of the total radioactivity in plasma [16]. Urinary excretion was the primary route of elimination of the absorbed dose in both rats and humans following IV RDV (mean values of 61% and 74%, respectively). This was also a major route of excretion following oral ODV (means of 33% and 58%, respectively) and almost entirely as GS-441524. Fecal excretion was also noted as an elimination route of radioactivity following IV RDV in both rats and humans, accounting for approximately 25% and 18% of the respective administered dose. Following oral ODV, means of 52% and 32%, respectively, of the administered dose in rats and humans were recovered in feces, primarily as GS-441524 (~85 to 98% of the total sample radioactivity, respectively). Deaminated nucleoside was observed as a minor metabolite in feces, at levels 5- to 10-fold lower, indicating minimal impact of this metabolic route on ODV bioavailability. Overall, GS-441524 was the most abundant systemic metabolite following IV RDV and oral ODV administration, and the absorbed dose was mainly cleared as GS-441524 via renal elimination.

## 4. Discussion

The optimization of nucleos(t)-ide analogs requires a careful balance to be maintained between PK properties and pharmacologic considerations for the target viral polymerase. It is crucial to appreciate that, similar to a prodrug, GS-441524 itself is not an active species but a metabolic precursor. Pharmacologically active GS-443902 can only be generated intracellularly through successive kinase-catalyzed metabolic steps, first from GS-441524 to the monophosphate, then diphosphate, and finally to the GS-443902-triphosphate metabolite. Therefore, the goal of nucleos(t)-ide prodrug design is ultimately to maximize the delivery and formation of active intracellular triphosphate inside cells susceptible to virus infection.

Two prodrugs were designed around GS-441524, namely, RDV and ODV, which solve different hurdles with respect to the noted limitations of GS-441524. RDV is a prodrug of the GS-441524-monophosphate (nucleotide) metabolite, while ODV is a 5’-ester prodrug of GS-441524 (nucleoside). In vitro, studies showed a significantly more favorable formation of active GS-443902 triphosphate following incubation with RDV compared to either GS-441524 itself or as ODV [11,17]. In vivo, the results obtained following IV RDV further confirmed this mechanism, with more efficient GS-443902 triphosphate formation observed in various tissues (e.g., PBMC and lung; Figure 5) compared to either IV GS-441524 or oral ODV.

Understanding that GS-441524-related nucleoside prodrugs (e.g., ODV) and nucleotide prodrugs (e.g., RDV) are different in nomenclature and in their design is essential, and the interpretation of PK data is not necessarily equivalent between the different prodrugs. As such, RDV was designed as a vehicle to efficiently deliver the GS-441524-monophosphate precursor inside cells and then utilize the anabolism advantage of the monophosphate to form GS-443902, which is kinetically favored over the nucleoside analog. In contrast, ODV was optimized to maximize the oral bioavailability of GS-441524, which is estimated at 41% to 50% following clinical ODV administration of a 350 mg oral tablet [16] vs. <15% estimated from human PK reported following consumption of an oral solution (55 °C) of GS-441524 [46], and thereby increase the concentration of GS-441524 inside cells. Collectively, RDV and ODV are both prodrugs, but they differ in their design because they deliver different GS-443902 precursors into cells.

The efficacy relationship of the two different prodrug approaches was studied in NHP models of infection. IV GS-441524, as well as the two prodrugs administered daily, were found to have similar antiviral efficacy in vivo in nonhuman primate models of SARS-CoV-2 infection [11,19]. The administration of the nucleoside GS-441524, as well as both prodrugs, results in rapid and broad distribution, as demonstrated in rats by [^14^C]QWBA studies, as well as in monkeys in the select tissues analyzed. MARG analysis following IV [^3^H]RDV or [^3^H]GS-441524 in rats also showed no discernible difference in lung and trachea sub-regional distribution, indicating that RDV or GS-441524 (via ODV) would reach and likely form GS-443902 in virus-infected cells lining the respiratory tract epithelium. To the best of our knowledge, this is the first report showing either RDV or GS-441524 distribution into respiratory tract cells. However, the IV RDV tissue uptake/distribution, both in exposure and extent, is higher compared to a molar dose equivalent of oral ODV, which justifies the requirement for higher ODV molar doses in efficacy experiments.

The PK studies presented and discussed highlight the rapid appearance of GS-441524 in vivo in plasma, as the major metabolite observed following the administration of either IV RDV or oral ODV (Figure 5A). However, the plasma PK of this nucleoside metabolite is distinguishable between ODV and RDV. Plasma GS-441524 from RDV is characterized by a lower exposure profile (C_max_ and AUC_(0-t)_) and a longer terminal half-life than that from ODV. An obvious question then would be, why is the estimated terminal elimination half-life different? If GS-441524, following IV RDV, enters and is then primarily cleared from the plasma (or systemic circulation) compartment in the same manner as oral ODV, the terminal half-life of GS-441524 would be expected to be the same. The half-life difference suggests they are not equivalent, and the collection of in vitro experimental data begins to elucidate how distinct metabolic pathways between these two prodrugs, RDV and ODV, lead to this in vivo GS-441524 PK difference.

While in vitro plasma incubations show similar instability for both ODV and RDV that are consistent with plasma esterase expression [47], the similarities and eventual path to GS-441524 diverge. Indeed, RDV and ODV include prodrug design efforts to take advantage of their intended routes of administration. Following IV administration, the improved permeability of RDV allows for the majority of the intact prodrug to more readily enter tissues, including virally infected cells, despite its transient plasma exposure. Thereafter, the metabolic route is more complex and substantially different than the simple cleavage of ODV, as RDV irreversibly passes through an intermediate alaninyl metabolite, i.e., GS-704277, en route to the GS-441524-monophosphate. Histidine triad nucleotide binding protein 1 (HINT1; [48]) is an intracellular enzyme primarily responsible for cleaving the phosphoramide (P-N) bond to form the GS-441524-monophosphate metabolite. Without access to intracellular HINT1, and consistent with P-N bond chemical stability [49], GS-704277 itself is not significantly metabolized in cyno or human plasma incubations, as shown herein (Figure 2C,D). Consequently, no detectable, direct formation of GS-441524 is anticipated to occur in the plasma compartment from alaninyl intermediate GS-704277, as shown in Figure 1. From the GS-441524-monophosphate metabolite, successive intracellular anabolism leads to GS-443902 formation and its accumulation in tissues, governed by its intracellular half-life. The mechanisms contributing to the effective intracellular half-life of GS-443902 include affinity and/or capacity for kinase activation versus dephosphorylation of the GS-441524-phosphorylated species and the effective rate of cellular release of GS-441524. The cellular release of GS-441524 is thought to be primarily governed by its substrate properties for ENTs, given its limited passive permeability properties [50,51,52]. The GS-441524 plasma half-life following IV RDV administration is, therefore, reflective of mechanisms governing the intracellular half-life of GS-443902. Consequently, plasma GS-441524 observed following RDV administration displays formation-limited flip–flop kinetics, governed primarily by its “absorption” into plasma or, specifically, its release from the intracellular compartment. Additionally, in consideration of alternate routes of administration, RDV was markedly unstable in hepatic S9 fractions, which is typical of phosphoramidate prodrugs [53]. With a predicted human hepatic extraction >0.9, oral administration would likely preclude systemic delivery of RDV. In fact, a similarly extensive hepatic extraction for RDV in cyno was predicted in vitro and, indeed, was confirmed to have negligible oral bioavailability (<1%) in vivo [11,17].

For oral ODV, the esterases first encountered during absorption, located within GI enterocytes—as evidenced in both GI S9 incubations (Figure 3) and the in vivo portal vein study (Figure 6)—extensively convert ODV to GS-441524, which rapidly enters systemic circulation. Any small amount of ODV that escapes GI intact is also rapidly metabolized to GS-441524 both extra-hepatically (e.g., by plasma hydrolases) as well as in the liver. These data also establish that in vivo, the main organ for treatment, e.g., the lung in the case of SARS-CoV-2, is not exposed to the intact ODV prodrug, thereby nullifying any improved cell culture antiviral activity for the prodrug ODV compared to GS-441524 (Figure 1). Systemically circulating GS-441524 is then primarily cleared by renal excretion, as demonstrated in nonclinical species (as described in Section 3.2.5 and Section 3.2.7) and in humans [16]. A small portion of GS-441524 in plasma is still able to enter cells, likely facilitated by nucleoside transporters (described in Section 3.1.3), where it is successively phosphorylated to GS-443902. However, since oral ODV-delivered GS-441524 forms substantially lower GS-443902 levels in tissues (compared to a molar equivalent dose of RDV), the intracellular source of GS-441524 released back into plasma, as described in the preceding paragraph, is quantitatively negligible. Consequently, no discernible GS-443902-derived contribution of GS-441524 is observed in the plasma exposure profile, where its elimination is primarily governed by rapid renal excretion. It can also be inferred from animal studies that showed equivalent IV RDV and oral ODV efficacy, and as shown in human PK studies, that despite the difference in intracellular metabolism efficiency to form GS-443902, efficacious levels of GS-443902 can be safely generated by increasing molar doses of oral ODV.

With a mindset towards the clinical testing of a new drug, it is often necessary to establish some PK-PD/efficacy relationships in nonclinical models to help determine the appropriate dosing regimen for efficacy in humans. In most instances, plasma exposures in animal models of an “active species” can be used to model target levels/exposures in the clinic. In the case of nucleosides or their prodrugs, where the active species is a downstream metabolite inside cells, it is often more challenging, and caution should be applied when considering systemically observed entities as a basis of dose and/or regimen projections. In the two prodrugs developed for GS-441524, the main systemic metabolite in both cases is GS-441524 (Figure 5). However, following IV RDV, plasma GS-441524 is neither the sole nor primary entity responsible for efficient GS-443902 formation in tissues that drives efficacy, as some have postulated. This is in contrast to oral ODV, where plasma GS-441524 is the primary entity responsible for GS-443902 formation inside cells. As a result, at any given point in time, the GS-443902 concentrations inside cells relative to GS-441524 plasma exposure from the delivery of the two different prodrugs are not equivalent, as clearly illustrated in Figure 5. This disparity is also supported by the fact that the plasma exposures of GS-441524 are very different when efficacy is similar in the animal models. Simply stated, the observed plasma concentrations of the main metabolite produced by both prodrugs, GS-441524, cannot be considered the same when projecting human dosing regimens.

An accepted method of human dose prediction for many antivirals is to apply the inhibitory quotient (IQ), where IQ represents a ratio of a compound’s plasma trough concentration in vivo to a measure (e.g., EC_95_) of its plasma protein binding-adjusted in vitro potency in a cell-based assay, together with an “x”-fold multiplier. The x-fold multiplier is necessary to establish and maintain efficacy, deal with interindividual variability in PK, prevent viral breakthrough, and control potential resistance mutations for that class of inhibitors. This method has the advantage that it can be used for clinical dosing projections in the absence of animal model data. For example, IQ is routinely applied to guide human dose projections for small-molecule, direct-acting HIV inhibitors (e.g., protease inhibitors, non-nucleoside reverse transcriptase inhibitors; [54,55,56]). However, applying plasma-based IQ method to nucleos(t)-ide reverse transcriptase inhibitors (NRTIs) and their prodrugs to predict doses mandates caution, largely owing to the necessary metabolism that occurs inside cells to form the active metabolite. Measurements of the active metabolite in vivo within an appropriate target tissue, instead of a plasma metabolite, are not trivial and bring along a multitude of challenges, including the ability to effectively and consistently collect, process, and accurately analyze target tissues [57,58,59,60] and potential species differences [61,62,63,64]. This is exemplified by the experiments reported herein, where lung tissue samples can be isolated and assessed; however, GS-443902 concentrations in only virally infected cells are beyond our current methods. For these reasons, a classical IQ approach was not favored for IV RDV or oral ODV human projections.

Given the different pathways to GS-443902 formation for ODV and RDV, it was necessary to use the nonclinical PK-PD/efficacy datasets for IV RDV and oral ODV independently to estimate their human efficacious doses and dosing regimens. For IV RDV, a dose regimen utilizing a 10 mg/kg loading dose followed by 5 mg/kg maintenance doses demonstrated efficacy in monkey models of SARS-CoV-2 infection [19,65,66] as well as in a number of filovirus models [17,25,67,68]. The corresponding human equivalent dose based on body surface area yields a 200 mg loading dose and 100 mg maintenance doses, respectively. Supporting this scaling approach, RDV systemic exposures in healthy monkeys (at 5 mg/kg IV infusion) were similar to the human exposures at 100 mg, with noted PK dose proportionality supporting extrapolation to the 200 mg loading dose [44]. In addition, PBMC concentrations of GS-443902, as a more readily sampled surrogate for respiratory tissue concentrations, were also similar between monkeys and humans [44] at the noted equivalent doses. No consideration was given to monkey or human systemic exposures of GS-441524 for the IV RDV human dose projections.

In contrast, GS-441524 plasma exposures in animal efficacy models [11,19] were used to predict the human efficacious dose regimen for oral ODV [16]. No intact prodrug was detected in plasma because of its rapid breakdown pre-systemically; hence, the only metabolic species to use for projections were the parent nucleoside or GS-443902 concentrations. Doses for the pivotal oral ODV monkey SARS-CoV-2 efficacy study were chosen to bracket the GS-441524 systemic exposures first established in the IV GS-441524 (20 mg/kg) AGM efficacy model [19]. Equivalent efficacy following IV RDV and relatively higher molar doses of oral ODV was observed despite the difference in intracellular metabolism efficiency to form GS-443902, inferring that efficacious levels of GS-443902 can be generated by increasing molar doses of oral ODV. GS-441524 plasma exposure at the efficacious oral ODV dose (at 60 mg/kg, once-daily) in monkeys was projected to be achieved with a clinical regimen of ODV at 350 mg–400 mg dosed twice daily, with an ultimate choice of 350 mg twice daily for phase 3 testing [15].

## 5. Conclusions

Both IV RDV and oral ODV are prodrugs of the intracellular triphosphate of the nucleoside analog GS-441524, which exhibit broad-spectrum antiviral activity. RDV and ODV show distinct metabolism and in vivo systemic PK profiles that, when combined, formulate a complete picture (Figure 1) of the metabolic fate in vivo of each prodrug. Importantly, we demonstrate, for the first time, that the plasma GS-441524 observed following IV RDV administration is not directly formed. Instead, its associated plasma kinetics is governed by the intracellular release of GS-441524 from the intact amidate prodrug. Conversely, we show, using a portal vein study, that orally administered ODV is predominantly metabolized to GS-441524 in GI tissue during absorption. GS-441524 then circulates systemically and distributes into tissues, where it is converted to its active triphosphate metabolite, while renal excretion governs a more rapid elimination of systemically circulating GS-441524. Moreover, using radiolabeled compounds, we demonstrate, for the first time, the delivery of GS-441524 and metabolites to specific layers of lung tissue. Despite intracellular conversion to the active triphosphate being kinetically faster and more efficient from systemic RDV than from GS-441524 following oral ODV administration, animal and human PK studies suggest this difference can be safely compensated by increasing doses of oral ODV. ADME studies also demonstrate rapid and broad tissue distribution of prodrug and/or metabolites from both prodrugs, supporting potential utility across multiple viruses with diverse tissue tropisms.

## Figures and Tables

**Table 1:** Summary of physicochemical, metabolic, and transporter characteristics of RDV, ODV, and primary circulating metabolite(s) ^a^.

Assay		RDV	GS-704277	GS-441524	ODV
**Solubility** (PBS; µg/mL)		40.1	ND	26.5	~1000
**LogD**		2.5	−10 ^b^	<0.3	0.9
**Permeability** (MDCK A-to-B; 10^−6^ cm/s)		1.32	ND	0.16	1.8
**Plasma protein binding** ^c^ (% free)		6–14	95–100	85–99	56–76
**Plasma stability** ^c^		Moderately Stable (unstable in mouse, rat)	Stable ^d^	Stable	Unstable (stable in dog)
**S9 Stability** ^c^	**Intestinal**	ND	ND	Stable	Unstable
	**Hepatic**	Unstable	ND	Stable	Unstable
**Transporter substrate**		OATP1B1/P-gp	OATP1B1/3	BCRP/P-gp ENT1/2/CNT3	BCRP/P-gp
**Enzyme hydrolysis** **phenotype** (f_m_)		CatA (10%) CES-1 (80%)	HINT1	ND	CES-1/2

Abbreviations: BCRP: breast cancer resistance protein; CatA: cathepsin A; CES: carboxylesterase; CNT: concentrative nucleoside transporter; ENT: equilibrative nucleoside transporter; f_m_: fraction metabolized; HINT: histidine triad nucleotide binding protein; MDCK: Madin–Darby canine kidney cell line; ND: not determined; OATP: organic anion transporting polypeptide; P-gp: P-glycoprotein; PBS: phosphate-buffered saline. ^a^ select data previously reported [9,10,11,39,40]; ^b^ calculated value; ^c^ assessed in mouse (except S9), rat, dog, monkey, and human; ^d^ assessed in monkey and human.

**Table 2:** Plasma pharmacokinetic parameters following the administration of IV RDV at 10 mg/kg as a 30-min infusion to cynomolgus monkeys.

	Mean ± SD PK Parameter (N = 3)			
	Plasma RDV	Plasma GS-704277	Plasma GS-441524	PBMC GS-443902
T_1/2_ (h)	0.40 ± 0.11	0.56 ± 0.01	16.1 ± 4.0	29.4 ± 3.0
T_max_ (h)	0.33 ± 0.13	0.58 ± 0	1 ± 0	4 ± 0
C_max_ (nM)	14,500 ± 2400	2200 ± 640	872 ± 163	54,600 ± 7400
AUC_0–t_ (nM·h)	7020 ± 1150	2640 ± 750	6340 ± 860	NC
Cl (L/h/kg)	2.40 ± 0.17	NA	NA	NA
V_ss_ (L/kg)	0.504 ± 0.175	NA	NA	NA

SD: standard deviation; NA: not applicable; NC: not calculated.

**Table 3:** Plasma GS-441524 and tissue GS-443902 pharmacokinetic parameters following a single IV administration of RDV or GS-441524 as a 30 min infusion or after oral ODV in cynomolgus monkeys (N = 3; mean ± SD).

Compound	RDV	GS-441524	ODV
Route	IV	IV	Oral
Test article dose (mg/kg)	10	20	11.6
GS-441524 equivalent dose (mg-eq/kg)	4.8	20	9.4
**Parameter**	**Plasma GS-441524** ^a^		
T_1/2_ (h)	NR ^b^	2.68 ± 0.07	2.62 ± 0.55
T_max_ (h)	1 ± 0	0.516 ± 0.058	1.67 ± 0.58
C_max_ (nM)	1400 ± 400	79,200 ± 6700	7570 ± 6070
AUC_0–24h_ (nM·h)	9070 ± 2080	124,000 ± 11,000	21,600 ± 11,000
	**PBMC GS-443902**		
C_max_ (µM)	46.8 ± 5.6	12.5 ± 1.6	3.01 ± 0.57
C_24h_ (µM)	31.0 ± 11.8	8.59 ± 0.52	1.90 ± 0.31
PBMC-to-plasma ratio ^c^	3.42	0.069	0.088
	**Lung GS-443902**		
C_24h_ (nmol/g)	1.39 ± 0.15	0.410 ± 0.087	*0.095* ^f^
Lung-to-plasma ratio ^d^	0.207 ± 0.032	0.003 ± 0.001	-
Lung-to-PBMC ratio ^e^	0.049 ± 0.016	0.048 ± 0.010	-

^a^ Plasma data following IV GS-441524 and oral ODV were previously reported [11]; ^b^ NR = not reported; T_1/2_ could not be characterized because of limited sampling through 24 h. ^c^ Ratio of PBMC GS-443902 C_24h_ to (plasma GS-441524 AUC_0–24h/_1000). ^d^ Ratio of lung GS-443902 C_24h_ to (plasma GS-441524 AUC_0–24h_/1000). ^e^ Ratio of lung GS-443902 C_24h_ to PBMC GS-443902 C_24h._^f^ Estimated 24 h lung concentration based on consistent lung to PBMC ratios, using a mean value of ~0.05.

**Table 4:** Qualitative assessment of radioactivity in distinct regions of lung or representative trachea tissue of male Sprague Dawley rats following a 30 min intravenous infusion of either [^3^H]GS-441524 or [^3^H]RDV at a nominal dose level of 10 mg/kg (3700 µCi/kg).

	GS-441524			RDV		
Lung Region Animal No.	101	102	103	201	202	203
Bronchus (cartilage)	++	+++	NP	NP	NP	NP
Bronchus (smooth muscle)	++	++	NP	NP	NP	NP
Bronchus (epithelium)	++	+++	NP	NP	NP	NP
Bronchioles	+++	+++	+++	+++	+++	+++
Alveoli wall	+++	+++	+++	+++	+++	+++
Alveoli sac	++	++	+	+	+	+
Blood vessels	+++	+++	+++	+++	+++	+++
Trachea (representative)	++	++	++	++	++	++

+++ = moderate levels of silver grains present; ++ = low levels of silver grains present; + = background levels of silver grains present; NP = region not present in sections.

## Data Availability

The original contributions presented in this study are included in this article. Further inquiries can be directed to the corresponding author.

## Abbreviations

The following abbreviations are used in this manuscript:

ADME	absorption, distribution, metabolism, and excretion
AGM	African green monkey
AUC	area under the curve
BCRP	breast cancer resistance protein
C_24h_	24 h concentration
CL	clearance
C_max_	maximum concentration
CNT	concentrative nucleoside transporter
ENT	equilibrative nucleoside transporter
eq	equivalent
GIS9	gastrointestinal S9 fraction
T_1/2_	half-life
HepS9	hepatic S9 fraction
HINT	histidine triad nucleotide binding protein
IQ	inhibitory quotient
IV	intravenous
LC-MS/MS	liquid chromatography–tandem mass spectrometry
MDCK	Madin–Darby canine kidney
MARG	microautoradiography
MDR	multidrug resistance
NRTI	nucleos(t)ide reverse transcriptase inhibitor
ODV	obeldesivir
OATP	organic anion transporter polypeptides
PBMC	peripheral blood mononuclear cell
P-gp	P-glycoprotein
PK	pharmacokinetics
PMSF	phenylmethylsulfonyl fluoride
QWBA	quantitative whole-body autoradiography
RDV	remdesivir
RSV	respiratory syncytial virus
RNA	ribonucleic acid
SARS-CoV	severe acute respiratory coronavirus
SD	standard deviation
V_ss_	steady-state volume of distribution
T_max_	time to maximum concentration
